# Supplementary material for: Indicator from the graph Laplacian of stock market time series cross-sections can precisely determine the durations of market crashes
Source: PLoS One. 2025 Jul 18;20(7):e0327391. doi: 10.1371/journal.pone.0327391 (PMC12273962; doi:10.1371/journal.pone.0327391)
Supplement: Attachment — Submitted filename: Response to Reviewers.pdf [file pone.0327391.s001.pdf]

Response to Reviewers

The comments by the Academic Editor did not point out specific flaws in our original manuscript, so we take it upon ourselves to improve the manuscript. The main changes are as follows:

(1) When we used $I_L(t)I_R(t)$ as the product signal, we end up with spurious peaks and 'echos'. Therefore, we modified the cross-filtering scheme to use $-I_L'(t)I_R'(t)$ as the product signal. This has the following advantage:

   a. no spurious peaks;
   b. no 'echos';
   c. no distortion, i.e., the product signal has the same shape as the signal itself.

   This modified cross-filtering scheme is simpler in behavior compared to the original cross-filtering scheme. Thus, we do not need to consider specific scenarios like what we did for the original cross-filtering scheme. This makes the Materials and Methods section significantly shorter.

   However, this modified cross-filtering method does have its own problems, which we described in the Results section and discussed in the Discussion section. As far as we can tell, the problem of the modified cross-filtering scheme is much less severe than the original cross-filtering scheme.

(2) We included the Nikkei 225 component stocks as a second cross section of stocks from a mature market (the first cross section being the component stocks of S&P 500), so that we can contrast two mature markets to two emerging markets.

(3) We rewrote the Results section and Discussion section based on the results obtained using the new cross-filtering scheme. These are now shorter than in the original manuscript.

We hope that with these revisions, the manuscript would be judged suitable for peer review.

Best regards,
Siew Ann Cheong
On behalf of Zheng Tien Kang and Peter Tsung-Wen Yen
