# Supplementary material for: Indicator from the graph Laplacian of stock market time series cross-sections can precisely determine the durations of market crashes
Source: PLoS One. 2025 Jul 18;20(7):e0327391. doi: 10.1371/journal.pone.0327391 (PMC12273962; doi:10.1371/journal.pone.0327391)
Supplement: Attachment — Submitted filename: Response_to_Reviewers_auresp_4.pdf [file pone.0327391.s006.pdf]

Response to Reviewers

<u>Reviewer #1:</u>

Comment: Thank you for your revisions. Your manuscript now demonstrates a coherent structure and a robust statistical approach that strongly supports the conclusions. The selection and explanation of your chosen markets is clear, and your writing style is sufficiently polished for publication. Overall, this version appears ready for acceptance.

Reply: We thank Reviewer 1 for the positive comments on the work done.

<u>Reviewer #5:</u>

Comment: I am satisfied with the review.
The authors have thoroughly addressed my questions.
I recommend publication.

Reply: We thank Reviewer 5 for the positive comments.

<u>Reviewer #7:</u>

Comment: I enjoyed the manuscript. The cross filtering is interesting, and I learned something new about the max spectral gap indicator. I have some minor points that should be addressed before publication.

Reply: We thank Reviewer 7 for the comments.

Comment: 1. "It is widely believed that daily market returns are normally distributed, with a mean close to zero, and standard deviation between 1%–2%" I would not say that this is still

widely believed. I would say, "a common assumption", or something of the sort. I think most know returns are heavy tailed.

Reply: We thank Reviewer 7 for the suggestion. We have changed the sentence mentioned to '*A common assumption is that the daily market returns are normally distributed, with a mean close to zero, and standard deviation between 1% - 2%*'.

Comment: 2. Literature review: The nonparametric multivariate changepoint literature, especially for changes in covariance should be at least pointed to, these methods can handle multiple series at once and some don't rely on parametric models. see e.g.,
- Matteson, David S., and Nicholas A. James. "A nonparametric approach for multiple change point analysis of multivariate data." Journal of the American Statistical Association 109.505 (2014): 334-345.
- Kelly Ramsay, Shojaeddin Chenouri, Robust nonparametric multiple changepoint detection for multivariate variability,
Econometrics and Statistics, 2023,ISSN 2452-3062, https://doi.org/10.1016/j.ecosta.2023.09.001.

Reply: We thank Reviewer 7 for the suggested articles. We have expanded the last sentence of paragraph 4 of the Literature Review section to include these references.

Comment: 3. "Second, some data may include 'NaN's, so we replaced them with '0's." You should not replace the prices with zeros - 0 is not a reasonable number for a stock price, and this artificially reduces the variance. At least replace with the previous day's price, or something more realistic

Reply: We thank Reviewer 7 for the critique. We checked our raw data for the four markets, and realized that Yahoo! Finance allows us to access historical prices of stocks up till the last changes in names or ticker symbols. For the SGX, we had to manually download the historical prices of 75

stocks from www.investing.com, whereas for TWSE, no problems were encountered. To make this clear, we have created a new second paragraph in the Data subsection.

Within our period of study, the number of stocks with missing price values and where they occur are shown in the table below for the four markets studied. When we computed the fractional returns using these price values, we ended up with ‘NaN’s whenever one of the prices is missing. It was at this point that we replaced the ‘NaN’s with ‘0’s. Stocks with more than 50% ‘0’s in their return time series were discarded, before we computed the Pearson cross correlations between stocks. We have revised the last paragraph of the Data subsection to make this clear.

	S&P 500	Nikkei 225	SGX	TWSE
No prices at all	40	0	0	0
Missing prices at the beginning of the time series	5	3	118	83
Missing prices at the end of the time series	4	0	37	656

If the missing price values are at the start of the time series, then these would be assigned to zero prices. When we compute the fractional returns, these zero prices would give us ‘NaN’s which we will have to re-zero anyway. For missing price values at the end of the time series, if we had set these to the last observed price, these would all give ‘0’s when we compute the fractional returns. Our approach and the approach suggested by Reviewer 7 would therefore generate the same results.

Comment: 4. "which is the negative product of slopes defined in the Materials and Methods section, we find both positive and negative persistent peaks." By slopes do you mean derivatives? The word slope does not appear before this. You should use consistent language. This is not clear. Put an equation number in Methods section and reference that.

Reply: We apologize to Reviewer 7 for the inconsistent usage of words. We have changed all occurrences of ‘slope’ to ‘derivative’, which is the term that we define in the Materials and

Methods section. We also follow Reviewer 7’s suggestion to include a displayed equation in the Materials and Methods section, and to reference it in the Results section.
