# Supplementary material for: Indicator from the graph Laplacian of stock market time series cross-sections can precisely determine the durations of market crashes
Source: PLoS One. 2025 Jul 18;20(7):e0327391. doi: 10.1371/journal.pone.0327391 (PMC12273962; doi:10.1371/journal.pone.0327391)
Supplement: Attachment — Submitted filename: Review comments.docx [file pone.0327391.s002.docx]

**Review comments**

The paper suggests a distinctive approach to predicting the onset and conclusion of market collapses by utilizing graph Laplacian and information cross-filtering with two-time windows. This innovative approach contributes a new viewpoint to the published literature on financial risk management. During the COVID-19 pandemic, the research examines stock market data, particularly on the S&P 500, SGX, and TWSE markets. This data is both timely and pertinent. The manuscript provides a comprehensive explanation of the relationship between market collapses and spectral gaps, as well as well-labeled graphs for the results. The discourse regarding persistent and non-persistent peaks is insightful and provides valuable insights into comprehending market collapses.
**Major Issues**
1. The introduction section must be revised to explicitly state the research void, the purpose of the study, and the actual contributions that your study made.

2. Adding a literature review section and theoretical foundation section could benefit a more precise understanding.

3. Although the method is theoretically sound, it may need to be more complex for non-technical financial analysts or risk managers to employ. Cross-filtering with two-time windows and spectral gaps necessitates substantial computational resources and specialized knowledge, which may restrict its practical application beyond academic circles.

3. The paper concentrates on a significant crash event (COVID-19) in only three stock markets (the S&P 500, SGX, and TWSE). Although these markets offer some contrast, a more comprehensive analysis encompassing a wider range of markets or distinct crash events could enhance the generalisability of the results. Additionally, the proposed method's performance in non-crash market conditions must be adequately addressed, which is essential for evaluating the method's reliability in various market environments. The analysis should be expanded to encompass multiple crash events, such as the 2008 financial crisis, and should consider a more diverse range of markets (e.g., emerging vs. developed, various geographies).

4. The authors recognize the issue of "spurious peaks" that arise when closely timed events overlap, complicating the interpretation of the results. Although this is addressed in the discussion, a more comprehensive examination of the methods to mitigate this issue is necessary. Proposing a more robust solution to address overlapping events could improve future iterations of the paper.

5. The paper predominantly relies on the maximum spectral gap of the graph Laplacian to signal market crashes. Although this is innovative, the complete complexity of financial markets may need to be adequately captured by relying on a singular indicator. The accuracy and robustness of predictions may be enhanced by integrating this with other statistical or machine learning-based methods. Provide additional details regarding the practical application of this method in real-time risk management. What are the essential computational instruments, and how can financial institutions incorporate these discoveries?

6. Despite the authors' assertion that defining market collapses as durations (rather than point events) is beneficial for risk management, they do not explore the practical implications of this approach for financial institutions. The study's relevance would be improved by a more in-depth examination of the practical implications and a comparison of this method with existing financial risk management tools. Expand the discussion on non-crash periods and propose potential remedies for the spurious peaks problem. Provide additional discussion.

7. The diction could be more lucid despite its academic nature. Readers who are not acquainted with the intricacies of graph theory or Laplacian spectra may find certain sections challenging due to their dense technical content. The paper would be more accessible to a broader audience if the language in these sections were simplified or additional context information was provided.

8. The structure of the paper needs to be revised, including the introduction, literature, methodology, results, discussion, implications, limitations, future directions, and conclusion. Another problem with this manuscript is that we need to see citations in the results and discussion sections. Citations from top academics can benefit you a lot while you are making arguments.
