# Supplementary material for: Indicator from the graph Laplacian of stock market time series cross-sections can precisely determine the durations of market crashes
Source: PLoS One. 2025 Jul 18;20(7):e0327391. doi: 10.1371/journal.pone.0327391 (PMC12273962; doi:10.1371/journal.pone.0327391)
Supplement: Attachment — Submitted filename: Response to Reviewers[88].docx [file pone.0327391.s003.docx]

Response to Reviewers

Reviewer 1

**Comment**: The researchers explained in the Data section that they chose the four financial markets because of their knowledge. This is an insufficient and unconvincing reason for academic research. No section was allocated to reviewing the literature, and this is a major weakness in the study that must be closed by reviewing several pieces of literature so that the theoretical aspect of the research is completed.

**Reply**: We thank Reviewer 1 for this comment. We have created a separate literature review section after the Introduction section of the manuscript and moved the literature review content previously in the Introduction section to the new section for better clarity. We pointed to this new literature review section in the organization paragraph of the Introduction section (last paragraph of the Introduction section), and explain why what we do is novel since no one has done this before.

In this new literature review section, we begin with a review of market states. We then survey previous works that support our assertion that market crashes are changes in phase with definite start and end points. In the next part of the literature review section, we survey the literature on change point methods, before proceeding to discuss the dangers of treating market phase/state change as a change point problem. Since we found no prior literature on this in financial markets, we review instead the literature on gene-detection algorithms. We noted that some gene-detection algorithms correctly identify the start and end points of genes only 60-80% of the time. The success rate for complex gene structures (complex sequence of transitions between market states) is even lower. Some algorithms find the start but not the end or the end but not the start of genes. There are also possibilities of false positives, i.e., that the start and end points detected are both wrong.

We then explicitly state the key research gaps in the current literature, which in our opinion is the lack of suitable change point detection methods to detect market crashes as a state with finite duration in a single step. Most change point detection algorithms identify this duration by detecting the start and the end in two steps. Only the hidden Markov Model (HMM) is capable of detecting the market crash as a separate hidden state in one step but HMMs are computationally very expensive. This explains why we use the cross-filtering method as a response to the primary research question (i.e., to find the market crash state in one step). As a secondary research question, we also want to know whether there are noticeable differences when we apply the method to mature markets vs emerging markets. This is why we chose 2 mature markets (S&P 500 and Nikkei 225) so that they can be compared against each other and 2 emerging markets (SGX and TWSE) for comparison against each other and comparison between mature and emerging markets.

Reviewer 2

**Comment**: All the comments has been addressed and they have worked on all the pin point part. Its glad to see such a paper.

**Reply**: We thank Reviewer 2 for the positive comments.

Reviewer 3

**Review comments**

The paper suggests a distinctive approach to predicting the onset and conclusion of market collapses by utilizing graph Laplacian and information cross-filtering with two-time windows. This innovative approach contributes a new viewpoint to the published literature on financial risk management. During the COVID-19 pandemic, the research examines stock market data, particularly on the S&P 500, SGX, and TWSE markets. This data is both timely and pertinent. The manuscript provides a comprehensive explanation of the relationship between market collapses and spectral gaps, as well as well-labeled graphs for the results. The discourse regarding persistent and non-persistent peaks is insightful and provides valuable insights into comprehending market collapses.

**Reply**: We thank Reviewer 3 for the careful reading for our manuscript and the positive comments.

**Major Issues**
1. The introduction section must be revised to explicitly state the research void, the purpose of the study, and the actual contributions that your study made.

**Reply**: We thank Reviewer 3 for this comment, which was also raised by Reviewer 1. We have now added a Literature Review section where we identify the key gaps in our present knowledge of market crashes. Our primary research question is on the direct identification of a market crash period. We also have a secondary research question on whether there are discernible differences between market crashes on mature vs emerging markets. As requested by Reviewer 3, we rephrased a sentence in the Introduction section to make clear that our contribution is the information cross-filtering method using two adjoining time windows.

2. Adding a literature review section and theoretical foundation section could benefit a more precise understanding.

**Reply**: We thank Reviewer 3 for this comment, which was also raised by Reviewer 1. We have since added the literature review section. As explained above, our main contribution in this manuscript is the cross-filtering method, instead of new theory on market crashes. This is why there is no additional theoretical foundation section since the cross-filtering method is already described in detail in the Materials and Methods section.

3a. Although the method is theoretically sound, it may need to be more complex for non-technical financial analysts or risk managers to employ. Cross-filtering with two-time windows and spectral gaps necessitates substantial computational resources and specialized knowledge, which may restrict its practical application beyond academic circles.

**Reply**: We thank Reviewer 3 for this comment. In this study, we apply the cross-filtering method onto the spectral gap indicator because we are confident that it is very sensitive to market crashes. In general, the method can be applied to other indicators, depending on the need and the purpose.

For a cross-section of 500-800 stocks, we believe the computational time is acceptable to practitioners. We performed a runtime analysis of the various subcomponents in our MATLAB code on a mid-class home laptop (Dell Inspiron 5570 with 8 GB memory, Intel 8^th^ Gen Core i7-8550U CPU, codes and data stored on a 1TB HDD, using default integrated Intel UHD 620 graphic card). By utilizing the tic-toc functions in the default MATLAB library to compute runtime, we found that the TDA computation using MATLAB’s graph() function took approximately 4.3 seconds for filtration parameter 1.6 while the graph Laplacian computation time is approximately 3.3 seconds (using MATLAB’s Laplacian() function). The longest computation time of 63.9 seconds is recorded in the process of finding the eigenvalues of the graph Laplacian matrix using MATLAB’s eig() function. The window-based information cross filtering took just 0.55 seconds for detecting the maximum spectral gap in both filtration parameters 1.6 and 1.8 where they are most likely to be found. We believe runtime that slightly exceeds one minute should be acceptable to all practitioners.

We have not included other “overhead” costs such as saving values into matrices/arrays, saving to .mat files for future reading and plotting the graphs in our computation time analysis above. We believe these can be optimized depending on user’s needs and may be hardware-dependent. We reckon that a typical commercial bank’s or investment bank's desktop workstation (and hedge fund’s or quant fund's high performance computing clusters) can easily bring the computation costs down way below the figures above with efficient programming techniques.

3b. The paper concentrates on a significant crash event (COVID-19) in only three stock markets (the S&P 500, SGX, and TWSE). Although these markets offer some contrast, a more comprehensive analysis encompassing a wider range of markets or distinct crash events could enhance the generalisability of the results. Additionally, the proposed method's performance in non-crash market conditions must be adequately addressed, which is essential for evaluating the method's reliability in various market environments. The analysis should be expanded to encompass multiple crash events, such as the 2008 financial crisis, and should consider a more diverse range of markets (e.g., emerging vs. developed, various geographies).

**Reply**: We thank Reviewer 3 for these comments. First, we would like to point out that we tested four markets. We agree with Reviewer 3 that we can learn more by testing the cross-filtering method on more markets and more crashes. This would deepen our insights into market crashes in general. However, this is not the aim of our manuscript, which is to introduce the cross-filtering method, which we tested on artificial time series data as well as the well-defined one-year time series data from the four stock markets, featuring the COVID-19 crash near the midpoint of the time series. We hope that Reviewer 3 will agree with us that this is an adequate demonstration of the power of the cross-filtering method. In future, we may undertake a more systematic study of different market crashes on the same market, as well as specific market crashes that can be seen across different markets.

4. The authors recognize the issue of "spurious peaks" that arise when closely timed events overlap, complicating the interpretation of the results. Although this is addressed in the discussion, a more comprehensive examination of the methods to mitigate this issue is necessary. Proposing a more robust solution to address overlapping events could improve future iterations of the paper.

**Reply**: We thank Reviewer 3 for this comment. However, we are worried that Reviewer 3 might have read version 1 of the manuscript instead of the more recently-submitted version 2. A spurious peak arising from closely-timed events was a serious problem in version 1 of the manuscript, but we have fixed this problem in version 2 of the manuscript by adopting a slope-based cross-filtering method.

5a. The paper predominantly relies on the maximum spectral gap of the graph Laplacian to signal market crashes. Although this is innovative, the complete complexity of financial markets may need to be adequately captured by relying on a singular indicator. The accuracy and robustness of predictions may be enhanced by integrating this with other statistical or machine learning-based methods.

**Reply**: We thank Reviewer 3 for this comment and agree that a single indicator may not be sufficient to capture the full complexity of financial markets. However, in this paper we are only interested in the periods of market crashes. So far, the maximum spectral gap is the most sensitive indicator we have seen on this state of the market. We agree with Reviewer 3 that a combination of methods could be used to more accurately determine the start and end of market crashes, in particular for risk management purposes. But we believe this should be future work.

5b. Provide additional details regarding the practical application of this method in real-time risk management.

**Reply**: We apologize to Reviewer 3 that we have only mentioned risk management in an abstract manner in our paper. Let us give a concrete suggestion here (which we also include in the revised manuscript) on how we can use information on the start and end of market crashes to manage risk. We do so not for high frequency traders who frequently make the most profits during market crashes, but for long-term investors who need to hold on to their investment portfolio for an extended period of time. For these investors, the key decision criterion would be the long-term growth prospect of the stocks they are holding on to. During a market crash, the prices of these stocks may deviate significantly from their fundamental values, making it risky to buy or sell such stocks for the purpose of portfolio rebalancing. Long-term traders such as these should wait out the market crash, examine the changes in fundamental value, if any, before resuming trading activities.

5c. What are the essential computational instruments, and how can financial institutions incorporate these discoveries?

**Reply**: We thank Reviewer 3 for this comment. The maximum spectral gap and the cross filtering is done in MATLAB but we understand that financial institutions are not in favour of the yearly subscriptions to keep their MATLAB versions up to date. The same computational tasks can be implemented in Python or Julia, both of which are free. We do not expect financial institutions to base all decision making on the findings reported here but can add these to their list of decision metrics.

6. Despite the authors' assertion that defining market collapses as durations (rather than point events) is beneficial for risk management, they do not explore the practical implications of this approach for financial institutions. The study's relevance would be improved by a more in-depth examination of the practical implications and a comparison of this method with existing financial risk management tools. Expand the discussion on non-crash periods and propose potential remedies for the spurious peaks problem. Provide additional discussion.

**Reply**: We thank Reviewer 3 for this comment, and apologize for alluding to the benefits for risk management but not actually outlining how this can be done. We have since added a few sentences in the Conclusion section on specific risk management practices that can be implemented by traders.

As for Reviewer 3’s suggestion to compare our method against existing financial risk management tool, using specific practical indicators (e.g., VaR, expected shortfall) as the performance of the methods, we believe that this is outside the scope of our manuscript, and based on the amount of work needed, would indeed be a manuscript all by itself.

Finally, let us address Reviewer 3’s comment on non-crash periods. We apologize that we did not make it clear in the manuscript, but the test of the cross-filtering method is not simply what it found for the COVID-19 crash and related market corrections, but also that it did not find sizeable signals during periods when there is no crash. We have since added a sentence to make this clear in the Results section.

7. The diction could be more lucid despite its academic nature. Readers who are not acquainted with the intricacies of graph theory or Laplacian spectra may find certain sections challenging due to their dense technical content. The paper would be more accessible to a broader audience if the language in these sections were simplified or additional context information was provided.

**Reply**: We thank Reviewer 3 for this comment and apologize that we have assumed an unnecessary high familiarity with graph theory in our readers. We have since added a figure in the Materials and Methods section explaining how we would get different networks at different scales, and how the graph Laplacian can be computed from the adjacency matrix at each scale.

We have also noticed a typographical error in the definition of the maximum spectral gap and have fixed it. With the aid of this additional figure, we also explained the circumstances under which a large spectral gap will appear.

8. The structure of the paper needs to be revised, including the introduction, literature, methodology, results, discussion, implications, limitations, future directions, and conclusion. Another problem with this manuscript is that we need to see citations in the results and discussion sections. Citations from top academics can benefit you a lot while you are making arguments.

**Reply**: We thank Reviewer 3 for this comment, but feel that the suggestion would lead to too many short sections at the end of the manuscript. Instead, we included the limitations of our method in the Discussion section, and also sketched implications and future directions in the Conclusion section. We will highlight these more clearly to readers, but hope that Reviewer 3 will agree with our decision to not have too many sections.

We also thank Reviewer 3 for the suggestion to add references in the Results and Discussion sections, to point readers to additional resources in the literature. We have done this to the best of our ability.

Reviewer 4

**Comment**: Review of “Indicator from the graph Laplacian of stock market time series cross sections can precisely determine the durations of market crashes”

PONE-D-24-21514R1
Accepted

**Reply**: We thank Reviewer 4 for this positive recommendation.

This paper delivers novel theoretical conclusions

**Reply**: We thank Reviewer 4 for the positive comment.

Introduction
clear

**Reply**: We thank Reviewer 4 for the positive comment that our introduction is written clearly.

Theoretical framework
sufficiently robust.

Research design
sufficiently robust.

Descriptive Statistics
The descriptive statistics is sufficiently robust.

Conclusions, Implications, and Recommendations
sufficiently robust.

**Reply**: We thank Reviewer 4 for the positive comments that our theoretical framework, research design, descriptive statistics, conclusions, implications and recommendations are robust.
