# Supplementary material for: Indicator from the graph Laplacian of stock market time series cross-sections can precisely determine the durations of market crashes
Source: PLoS One. 2025 Jul 18;20(7):e0327391. doi: 10.1371/journal.pone.0327391 (PMC12273962; doi:10.1371/journal.pone.0327391)
Supplement: Attachment — Submitted filename: review.docx [file pone.0327391.s004.docx]

Predicting the patterns of the stock market is always an important task. This paper presents an interesting perspective that is a valuable addition to the literature. One thing I feel the authors can add is the comparison with other existing methods/models. Even if it cannot be done and included in the current manuscript, it should be reviewed and included as future work. Some of the related methods can be seen below:

1. Shu M, Zhu W. (2022). Dissecting the 2015 Chinese Stock Market Crash. **Stat**; [https://doi.org/ 10.1002/sta4.460](https://doi.org/10.1016/j.physa.2021.126425)
2. Shu M, Song R, Zhu W. (2021). The ‘COVID’Crash of the 2020 US Stock Market. **The North American Journal of Economics and Finance**, 101497.
