# Supplementary material for: Indicator from the graph Laplacian of stock market time series cross-sections can precisely determine the durations of market crashes
Source: PLoS One. 2025 Jul 18;20(7):e0327391. doi: 10.1371/journal.pone.0327391 (PMC12273962; doi:10.1371/journal.pone.0327391)
Supplement: Attachment — Submitted filename: Response to Reviewers.docx [file pone.0327391.s005.docx]

Response to Reviewers

Reviewer #1:

**Comment:** The manuscript demonstrates significant improvements in addressing the previous comments; however, there are still areas that require minor revisions to enhance clarity and coherence.

**Reply:** We thank Reviewer 1 for the positive comment.

**Comment:** While the manuscript is technically sound overall, the authors should provide further elaboration on the choice and justification of the selected markets to strengthen the theoretical foundation and address potential gaps in the literature.

**Reply:** We thank Reviewer 1 for the constructive comment. MSCI, which is an authoritative source in the finance industry, lists Hong Kong and Singapore as developed markets [1] (see pages 4 and 5 of the table in the link). However, we disagree with the classification of Singapore because traders on SGX don’t have the same level of sophistication as those in NYSE and the Tokyo Stock Exchange (TSE). Also, company valuations on the SGX are lower than similar companies listed on other markets. Overall, it is a much more illiquid market compared to the former two and has seen substantial delisting/privatization far outpacing new IPOs since the lates 2010s [2–6]. Our assessment of Singapore as an emerging market is corroborated by a recent study, showing that Singapore shares similar emerging markets attributes with other developing ASEAN markets [10].

We also reviewed works from various economics and finance journals and noted that different researchers have different opinions on the taxonomy of stock markets. Firstly, through clustering and centrality analysis of the minimal spanning tree (MST) of world stock market indices, it is found that throughout 2005 to 2014 US and the developed European markets are closely linked with one another. Emerging Asian markets are further apart and linked to the developed markets via the Japanese market. The developed Japanese market, together with the US and German markets, act as hubs in the network of world stock markets [7]. A separate dynamic correlation analysis suggested that Hong Kong and Japan both exhibited developed market characteristics [8]. The US market is generally regarded as the engine of growth for the whole world, thus it is natural to include it in our studies. We also chose the Japanese market as a contrasting developed market, because it is in Asia, and because the GDP of Japan is the third largest (formerly second largest) in the world.

Having included the Japanese market as one of the two developed markets, it is natural to include emerging markets from Asia as a contrast. In particular, the Asian Tigers of Hong Kong, Singapore, South Korea, and Taiwan would be very good choices because of their rapid ascent as emerging markets. In an analysis of events unfolding during the 2008 US subprime crisis, South Korea and Singapore are considered emerging markets [9]. In another study investigating the financial interdependence between markets, South Korea and Taiwan are listed alongside Mexico as among the more advanced emerging markets [10]. However, we realized that it is not necessary to include all four markets in our study, not least because of the classification of Hong Kong as a developed market. In general, strong correlations are observed between the Hong Kong and Singapore markets, as seen during the 1997 Asian Financial Crisis [8] and their relative positions in the MST analysis of the recent COVID-19 crash [7,11,12]. Because of their similar industry mix, and focus on the semiconductor industry, we also see that Taiwan is the nearest neighbour of South Korea in the MST [7,11,12]. Therefore, we chose Singapore from the Hong Kong-Singapore pair and Taiwan from the South Korea-Taiwan pair of emerging markets as contrasts to the US and Japanese markets.

Through the literature review above, we are confident that Taiwan and Singapore are correctly classified as emerging markets (though more advanced than other emerging markets) while US and Japan are truly developed markets, in agreement with other experts.

**Comment:** Additionally, the manuscript contains sections that are unclear or written in a way that hinders readability, and revisions are recommended to improve sentence structure, fix minor grammatical errors, and enhance the flow of ideas. Overall, I recommend Minor Revisions to address these points and enhance the overall quality of the manuscript.

**Reply:** We apologize to Reviewer 1 still finds our writing difficult to follow. We have been very careful in our revision, in particular we have systematically rephrased sentences that sound ambiguous to readers. We are happy to further improve the readability of our manuscript, and therefore we have utilized several language checker tools such as ChatGPT and LanguageTool extension on Firefox to check for ambiguity and grammatical errors. The ChatGPT language checker function is integrated with Overleaf. Potential errors are underlined in yellow, as shown below:

As the Overleaf built-in checker function provides limited number of corrections and requires a premium subscription for full functionality, we also tried with the free LanguageTool extension on Firefox (also available on Google Chrome) to check for errors, as shown below:

Suggested correction of names of people and places in both tools are ignored as they are proper nouns, as shown below:

We are hopeful that with the thorough analysis of the manuscript with both language checker tools, we have addressed concerns on language issues from Reviewer 1. In case we overlooked and missed any sections, we hope to hear back from Reviewer 1 on the particular sections that hinder readability.

Reviewer #5:

**Comment:** Predicting the patterns of the stock market is always an important task. This paper presents an interesting perspective that is a valuable addition to the literature.

**Reply:** We thank Reviewer 5 for the positive comments on the works done.

**Comment:** One thing I feel the authors can add is the comparison with other existing methods/models. Even if it cannot be done and included in the current manuscript, it should be reviewed and included as future work. Some of the related methods can be seen below:

1. Shu M, Zhu W. (2022). Dissecting the 2015 Chinese Stock Market Crash. **Stat**; [https://doi.org/ 10.1002/sta4.460](https://doi.org/10.1016/j.physa.2021.126425)
2. Shu M, Song R, Zhu W. (2021). The ‘COVID’ Crash of the 2020 US Stock Market. **The North American Journal of Economics and Finance**, 101497.

**Reply:** We thank Reviewer 5 for the suggestions. We have added two new paragraphs on market crash predictions to the Literature Review section. The first paragraph is on prediction methods involving the use of Schefferian early warning indicators (SEWIs). SEWIs include increased variance, skewness, and kurtosis, as well as critical slowing down and spectral reddening, along with others like flickering and patchiness [13–17]. In their reviews [18,19], Scheffer et al. explained the critical slowing down phenomenon originated from the underlying dynamical systems. Whenever a complex system approaches a critical point or tipping point, it will take longer to return back to its equilibrium after perturbations. This universal behaviour precedes all critical transitions, thus the SEWIs can be used to identify and predict regime shifts in financial markets. Much inspired by the existing works, we also studied the US housing market crash during 2004 to 2008 [20,21], and more recently on high-frequency FOREX time series [22], using the SEWIs.

In the second paragraph, we described the log-periodic power law (LPPL) method, which is used by many more groups for the prediction of market crashes. This method was developed independently by two groups of people, namely, Feigenbaum et al. [23,24] and Sornette et al. [25]. Through fitting the financial time series to power laws with log-periodic decorations, we can estimate how far away is the crash (or any other extreme events) from the current time, the degree of super exponential growth of the current market state and the angular frequency of the log-periodicity [26–34]. In the figure below (but not in the manuscript), we show three successful LPPL fits to the Nasdaq composite index, the S&P 500 index and the Nikkei 225 index. The critical times $t_{c}$ are shown within the plots themselves.

| Index | LPPL Plots |
| --- | --- |
| Nasdaq Composite Index |  |
| S&P 500 Index |  |
| Nikkei 225 Index |  |

It is important to note that LPPL provides insights on a single time series only. Hence, underlying cross-correlations changes among index constituents will not be captured by the LPPL method.

Reviewer #6:

**Comment:** Thanks for giving me an opportunity to review the manuscript entitled “Indicator from the graph Laplacian of stock market time series cross sections can precisely determine the durations of market crashes”. This manuscript proposed the information cross-filtering method to determine the start and end points of market crashes.

**Reply:** We thank Reviewer 6 for the review.

**Comment:** I am concerned that the validation of method is lack of convincing, as only the COVID-19 crash was analyzed. The COVID-19 crash is a unique case of a crash that differs significantly from previous well-known market crashes, such as 2000 stock market crash. It is recommended to use different historical crash events to validate this method.

**Reply:** We thank Reviewer 6 for the suggestion. Since we worked with market cross-sections (including the whole of SGX and TWSE), the heavy computational load limited out initial focus to the COVID-19 crash period. We have since performed additional analysis on the 2008 Global Financial Crisis for the S&P 500 components and Nikkei 225 components and described our findings in the Discussion section, so as to not complicate the story-telling in the Results section.

We now believe that the complex peak structures seen in Fig. 5 and Fig. 6 (for COVID-19), as well as Fig. 7 and Fig. 8 (for the Global Financial Crisis) are the results of markets digesting a sequence of exogenous shocks generated by government interventions. We believe the simple peak structures seen in Fig. 3 and Fig. 4 for COVID-19 are the consequences of a lack of coherent government responses to the pandemic. We wish to thank Reviewer 6 for suggesting us to inspect other market crashes, pointing us to this alternative but more compelling interpretation of our results. We have changed part of the Conclusion to reflect this new understanding.

***References***

1.	Developed Markets Indexes | MSCI Indexes. [cited 16 Feb 2025]. Available: https://www.msci.com/indexes/group/developed-markets-indexes?index-sort=aToZ

2.	Making the Singapore market great again. In: The Edge Singapore [Internet]. 17 Oct 2024 [cited 16 Feb 2025]. Available: https://www.theedgesingapore.com/news/markets/making-singapore-market-great-again

3.	Chakraborty R. Pace of SGX delistings may not slow down in 2025: analysts. In: The Business Times [Internet]. 6 Jan 2025 [cited 16 Feb 2025]. Available: https://www.businesstimes.com.sg/companies-markets/pace-sgx-delistings-may-not-slow-down-2025-analysts

4.	Ruehl M, Lockett H. Singapore exchange suffers run of delistings. Financial Times. 17 Jun 2020. Available: https://www.ft.com/content/35057281-2c33-42f5-a211-c458731f97fd. Accessed 16 Feb 2025.

5.	Singapore Braces for More Delistings Even After Rule Fix. Bloomberg.com. 24 Jul 2019. Available: https://www.bloomberg.com/news/articles/2019-07-24/singapore-braces-for-delistings-to-continue-even-after-rule-fix. Accessed 16 Feb 2025.

6.	The incredible shrinking Singapore stock market. The Straits Times. 12 Feb 2019. Available: https://www.straitstimes.com/business/companies-markets/the-incredible-shrinking-singapore-stock-market. Accessed 16 Feb 2025.

7.	Wang G-J, Xie C, Stanley HE. Correlation Structure and Evolution of World Stock Markets: Evidence from Pearson and Partial Correlation-Based Networks. Comput Econ. 2018;51: 607–635. doi:10.1007/s10614-016-9627-7

8.	Chiang TC, Jeon BN, Li H. Dynamic correlation analysis of ﬁnancial contagion: Evidence from Asian markets.

9.	Dooley M, Hutchison M. Transmission of the U.S. subprime crisis to emerging markets: Evidence on the decoupling-recoupling hypothesis.

10.	Aloui R, Aïssa MSB, Nguyen DK. Global financial crisis, extreme interdependences, and contagion effects: The role of economic structure? J Bank Finance. 2011;35: 130–141. doi:10.1016/j.jbankfin.2010.07.021

11.	Memon BA, Yao H. The Impact of COVID-19 on the Dynamic Topology and Network Flow of World Stock Markets. J Open Innov Technol Mark Complex. 2021;7: 241. doi:10.3390/joitmc7040241

12.	Lavin JF, Valle MA, Magner NS. A Network‐Based Approach to Study Returns Synchronization of Stocks: The Case of Global Equity Markets. Xin B, editor. Complexity. 2021;2021: 7676457. doi:10.1155/2021/7676457

13.	Ismail MS, Noorani MSM, Ismail M, Razak FA, Alias MA. Early warning signals of financial crises using persistent homology. Phys Stat Mech Its Appl. 2022;586: 126459. doi:10.1016/j.physa.2021.126459

14.	Wang J, Zeng C, Han X, Ma Z, Zheng B. Detecting early warning signals of financial crisis in spatial endogenous credit model using patch-size distribution. Phys Stat Mech Its Appl. 2023;625: 128925. doi:10.1016/j.physa.2023.128925

15.	Diks C, Hommes C, Wang J. Critical slowing down as an early warning signal for financial crises? Empir Econ. 2019;57: 1201–1228. doi:10.1007/s00181-018-1527-3

16.	Song S, Li H. Early warning signals for stock market crashes: empirical and analytical insights utilizing nonlinear methods. EPJ Data Sci. 2024;13: 16. doi:10.1140/epjds/s13688-024-00457-2

17.	Bertschinger N, Pfante O. Early Warning Signs of Financial Market Turmoils. J Risk Financ Manag. 2020;13: 301. doi:10.3390/jrfm13120301

18.	Scheffer M, Bascompte J, Brock WA, Brovkin V, Carpenter SR, Dakos V, et al. Early-warning signals for critical transitions. Nature. 2009;461: 53–59. doi:10.1038/nature08227

19.	Scheffer M, Carpenter SR, Lenton TM, Bascompte J, Brock W, Dakos V, et al. Anticipating Critical Transitions. Sci Am Assoc Adv Sci. 2012;338: 344–348. doi:10.1126/science.1225244

20.	Tan J, Cheong SA. The regime shift associated with the 2004–2008 US housing market bubble. PLoS One. 2016;11: e0162140.

21.	Tan JPL, Cheong SSA. Critical slowing down associated with regime shifts in the US housing market. Eur Phys J B. 2014;87: 1–10.

22.	Wen H, Ciamarra MP, Cheong SA. How one might miss early warning signals of critical transitions in time series data: A systematic study of two major currency pairs. PLOS ONE. 2018;13: e0191439. doi:10.1371/journal.pone.0191439

23.	FEIGENBAUM JA, FREUND PGO. DISCRETE SCALE INVARIANCE IN STOCK MARKETS BEFORE CRASHES. Int J Mod Phys B. 1996;10: 3737–3745. doi:10.1142/S021797929600204X

24.	Feigenbaum JA, Freund PGO. Discrete Scale Invariance and the “Second Black Monday.” Mod Phys Lett B. 1998;12: 57–60. doi:10.1142/S0217984998000093

25.	Sornette D, Johansen A, Bouchaud J-P. Stock Market Crashes, Precursors and Replicas. J Phys I. 1996;6: 167–175. doi:10.1051/jp1:1996135

26.	Shu M, Song R, Zhu W. The ‘COVID’ crash of the 2020 U.S. Stock market. North Am J Econ Finance. 2021;58: 101497. doi:10.1016/j.najef.2021.101497

27.	Shu M, Zhu W. Dissecting the 2015 Chinese stock market crash. Stat. 2022;11: e460. doi:10.1002/sta4.460

28.	Song R, Shu M, Zhu W. The 2020 global stock market crash: Endogenous or exogenous? Phys Stat Mech Its Appl. 2022;585: 126425. doi:10.1016/j.physa.2021.126425

29.	Shu M, Zhu W. Detection of Chinese stock market bubbles with LPPLS confidence indicator. Phys Stat Mech Its Appl. 2020;557: 124892. doi:10.1016/j.physa.2020.124892

30.	Shu M, Zhu W. Real-time prediction of Bitcoin bubble crashes. Phys Stat Mech Its Appl. 2020;548: 124477. doi:10.1016/j.physa.2020.124477

31.	Geraskin P, Fantazzini D. Everything you always wanted to know about log-periodic power laws for bubble modeling but were afraid to ask. Eur J Finance. 2013;19: 366–391. doi:10.1080/1351847X.2011.601657

32.	Wheatley S, Sornette D, Huber T, Reppen M, Gantner RN. Are Bitcoin bubbles predictable? Combining a generalized Metcalfe’s Law and the Log-Periodic Power Law Singularity model. R Soc Open Sci. 2019;6: 180538. doi:10.1098/rsos.180538

33.	Wosnitza JH, Denz C. Liquidity crisis detection: An application of log-periodic power law structures to default prediction. Phys Stat Mech Its Appl. 2013;392: 3666–3681. doi:10.1016/j.physa.2013.04.009

34.	Zhang Q, Zhang Q, Sornette D. Early Warning Signals of Financial Crises with Multi-Scale Quantile Regressions of Log-Periodic Power Law Singularities. Gao Z-K, editor. PLOS ONE. 2016;11: e0165819. doi:10.1371/journal.pone.0165819
